# Supplementary material for: Visualizing inflammation with an M1 macrophage selective probe via GLUT1 as the gating target
Source: Nat Commun. 2022 Oct 10;13:5974. doi: 10.1038/s41467-022-33526-z (PMC9550770; doi:10.1038/s41467-022-33526-z)
Supplement: Supplementary file 1 — Supplementary information [file 41467_2022_33526_MOESM1_ESM.pdf]

Visualizing inflammation with an M1 macrophage selective probe via GLUT1 as the gating target

Heewon Cho,[a] † Haw-Young Kwon,[b] † Amit Sharma,[c] Sun Hyeok Lee,[a] Xiao Liu,[d] Naoki Miyamoto,[b] Jong-Jin Kim,[e] Sin-Hyeog Im,[c] Nam-Young Kang,[f] and Young-Tae Chang[a][b][d]

[a] School of Interdisciplinary Bioscience and Bioengineering, Pohang University of Science and Technology (POSTECH), Pohang 37673, Republic of Korea
[b] Center for Self-assembly and Complexity, Institute for Basic Science (IBS), Pohang 37673, Republic of Korea.
[c] Department of Life Sciences, Pohang 37673, Pohang University of Science and Technology (POSTECH)
[d] Department of Chemistry, Pohang University of Science and Technology (POSTECH)
[e] Department of Biology, Sunchon National University, Sunchon 57922, Republic of Korea
[f] Department of Convergence IT Engineering, Pohang University of Science and Technology (POSTECH), Pohang 37673, Republic of Korea

Correspondence E-mail: ytchang@postech.ac.kr

Contents of this SI file include:

• Supplementary Figure 1 to 23.

• Supplementary Table 1 to 3.

• Supplementary Methods including information on LC library and **CDr17** (Supplementary Figure 15 to 23, Supplementary Table 3).

List of Supplementary Figures:

Supplementary Table 1. The codes for LC library compounds.
Supplementary Fig. 1. The building blocks of fluorophores in LC library.
Supplementary Fig. 2. Characterization of polarized macrophages.
Supplementary Fig. 3. Selectivity index of M1 over M0 and M2 macrophages.
Supplementary Fig. 4. Selectivity index of M1 over M0 and M2 macrophages focusing on cy31 and cy51 fluorophores.
Supplementary Fig. 5. Selectivity confirmation of CDr17 with CDg16.
Supplementary Fig. 6. Concentration-dependent selectivity of 2-NBDG in RAW264.7,
Supplementary Fig. 7. Characterization of polarized macrophages from THP-1.
Supplementary Fig. 8. Selectivity of CDr17 in THP-1.
Supplementary Fig. 9. Concentration-dependent selectivity of 2-NBDG in THP-1.
Supplementary Fig. 10. Gene levels of SGLT family in differentiated macrophages from RAW264.7.
Supplementary Fig. 11. Immunochemistry confirms GLUT1 expression level in differentiated macrophages from RAW264.7.
Supplementary Fig. 12. Correlation between M1 polarization, CDr17, and GLUT1.
Supplementary Fig. 13. Target validation of CDr17 in CRISPRa-, and CRISPRi-GLUT1 model system.
Supplementary Fig. 14. CDr17 selectivity in M0, M1, and M2 macrophages derived from mouse peritoneal macrophages.
Supplementary Fig. 15. Tissue section images of CDr17 from acute inflammation-induced in vivo model.
Supplementary Fig. 16. CDr17 selectivity in CIA animals
Supplementary Fig. 17. Tracking the CDr17 signal in RA animals over time injection.
Supplementary Fig. 18. CDr17 specificity to M1 macrophages compared to other cell types in synovium tissues.
Supplementary Fig. 19. M1 macrophages have higher GLUT1 expression.
Supplementary Table 2. Sequences of primers.

Supplementary Methods

Supplementary Fig. 20. General schematic procedures of LC library
Supplementary Fig. 21. ^{1}H NMR spectrum of CDr17.
Supplementary Fig. 22. ^{13}C NMR spectrum of CDr17.
Supplementary Fig. 23. ^{1}H-^{1}H COSY of CDr17.
Supplementary Fig. 24. ^{1}H-^{13}C HSQC of CDr17.
Supplementary Fig. 25. ^{1}H-^{13}C HMBC of CDr17.
Supplementary Fig. 26. HPLC and mass chromatogram of CDr17.
Supplementary Fig. 27. HR-MS spectrum of CDr17.
Supplementary Fig. 28. Optical properties of CDr17 in DMSO
Supplementary Table 3. Characterization and purity determination of LC compounds by LC-MS.

Supplementary Results

Supplementary Table 1. The codes for LC library compounds

Carbohydrates		2	3	4	5	6	7	8	9	10	11
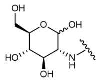	**A**	c1	c2	c4	n1	n2	d1	d2	f1	f2	b1
	**B**	b2	b3	b4	b5	b6	cy31	cy32	cy51	cy52	cy71
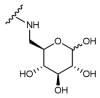	**C**	c1	c2	c3	c4	c5	n1	n2	d1	d2	f1
	**D**	b1	b2	b3	b4	b5	cy31	cy32	cy51	cy52	cy71
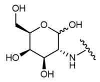	**E**	c1	c2	c3	c4	c5	n1	n2	d1	d2	f1
	**F**	b1	b2	b3	b4	b5	cy31	cy32	cy51	cy52	cy71
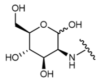	**G**	c1	c2	c3	c4	c5	n1	n2	d1	d2	f1
	**H**	b1	b2	b3	b4	b5	cy31	cy32	cy51	cy52	cy71

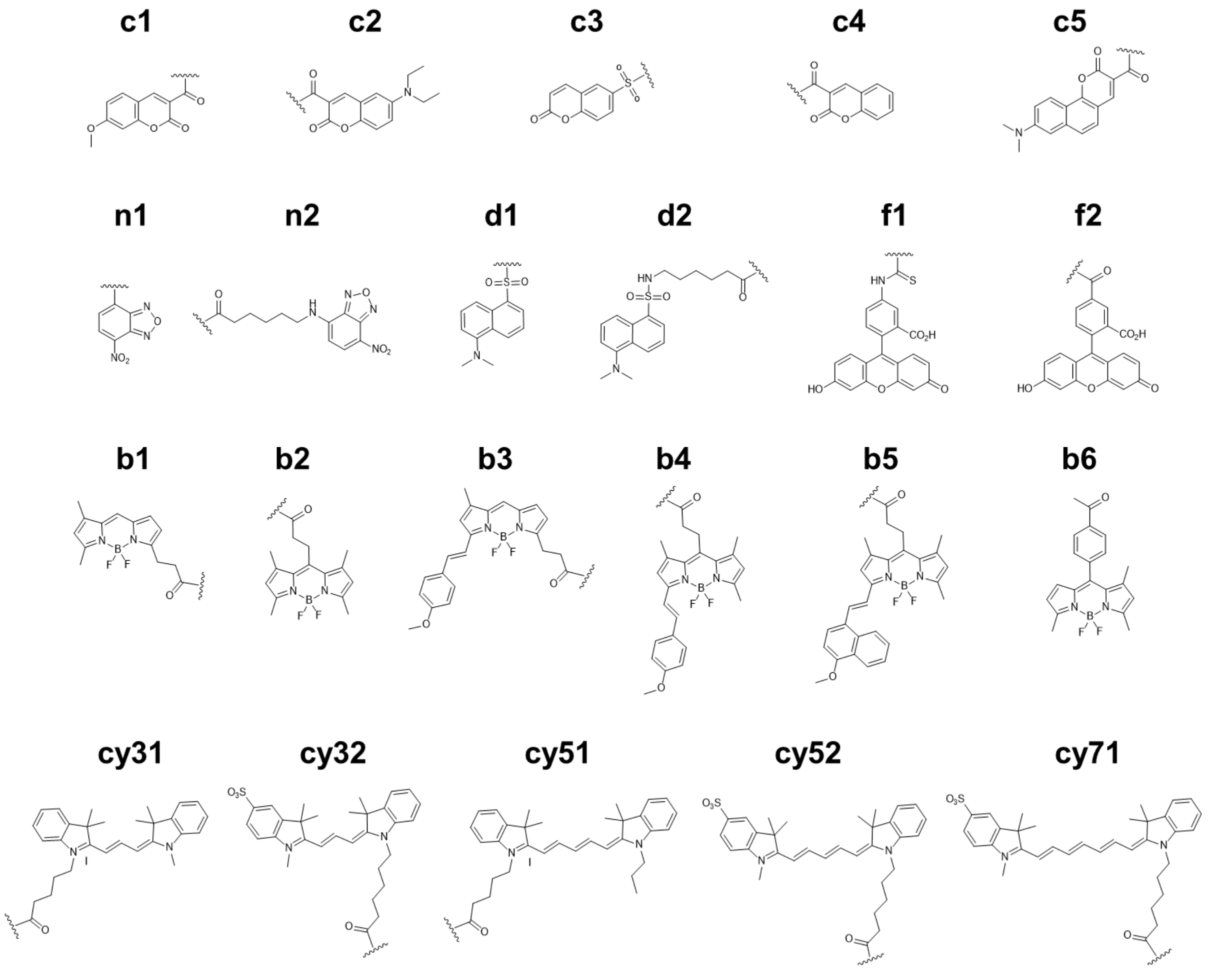

Supplementary Fig. 1. The building blocks of fluorophores in LC library.

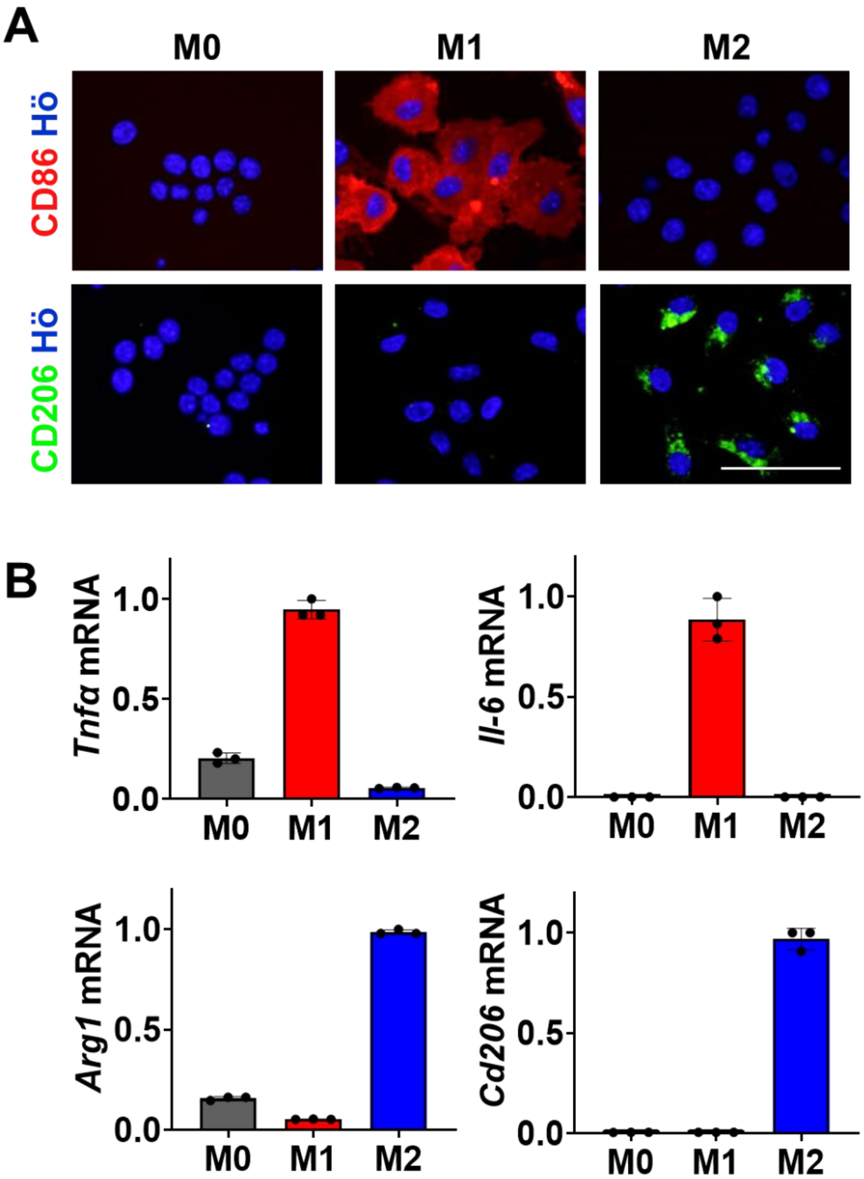

Supplementary Fig. 2. Characterization of polarized macrophages from RAW264.7 cell by A) images and B) gene expression of specific biomarkers. All the images were acquired at 40x magnification, and the scale par represented 50 μm. Data pooled from three individual experiments. Data are analyzed with 3 samples over 3 independent experiments. All bar graphs show the mean ± SD (n=3). Source data are provided as a Source data file.

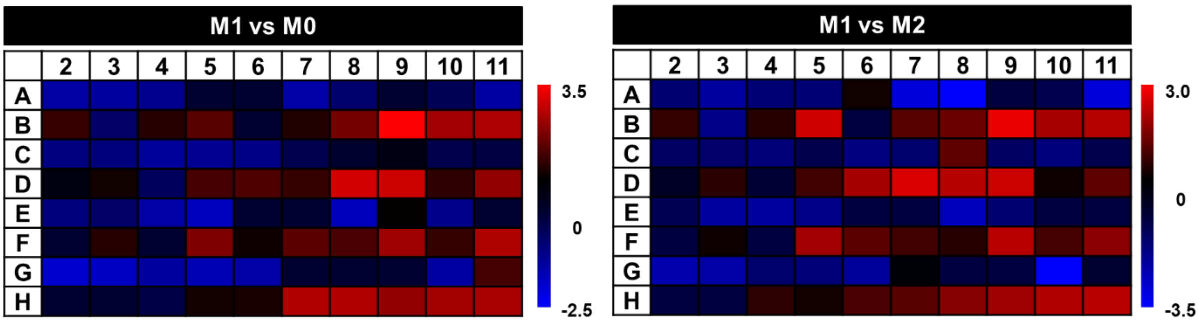

$$\text{Selectivity index} = \frac{F(M1)-F(M0\ \text{or}\ M2)}{\sigma(M1)+\sigma(M0\ \text{or}\ M2)}$$

Supplementary Fig. 3. Selectivity index of M1 over M0 and M2 macrophages. After screening M0, M1, and M2 macrophages with LC library, the fluorescent intensity was measured. With statistical value, the selectivity index was drawn, and expressed by a heat map. F: Fluorscent intensity; σ : standard deviation.

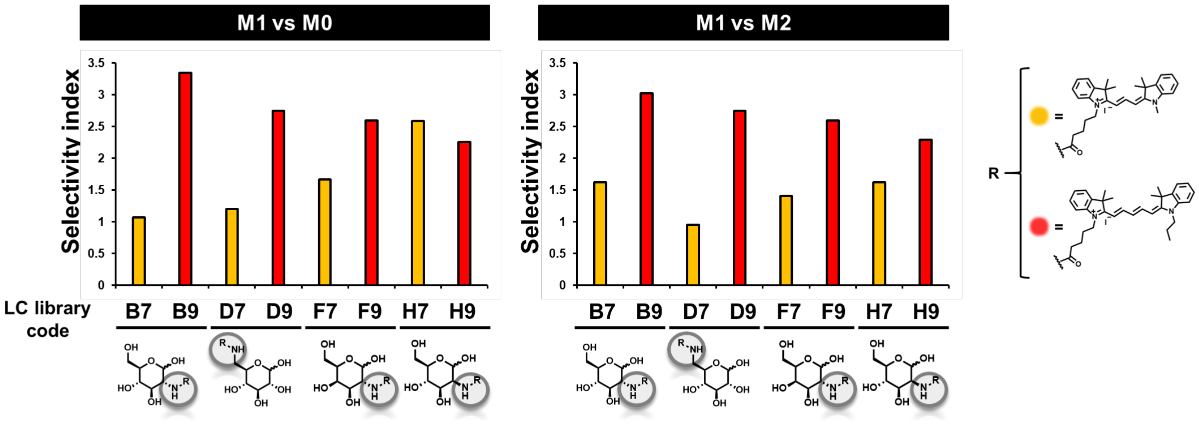

Supplementary Fig. 4. Selectivity index of M1 over M0 and M2 macrophages focusing on cy31 and cy51 fluorophores.

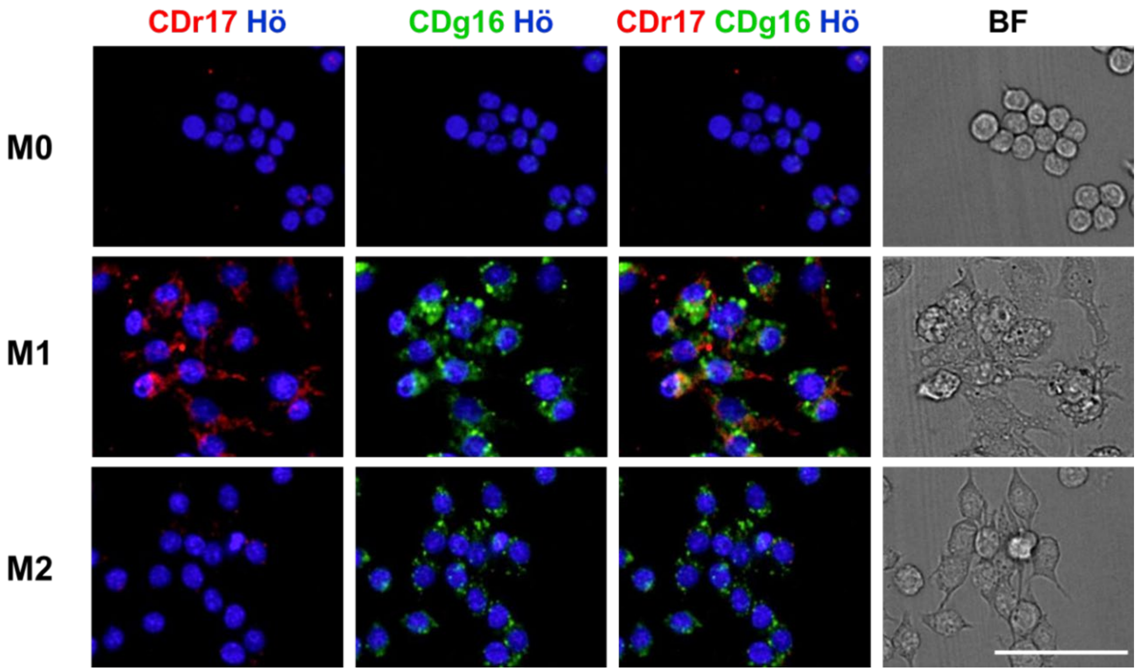

Supplementary Fig. 5. Selectivity confirmation of **CDr17** with **CDg16**. M0, M1, and M2 macrophages from RAW264.7. The cells were stained with 1 μM of **CDr17** (30 min) and **CDg16** (1 h). All the images were acquired at 40x magnification. Each three experiment was repeated independently with similar results. Scale bar, 50 μm.

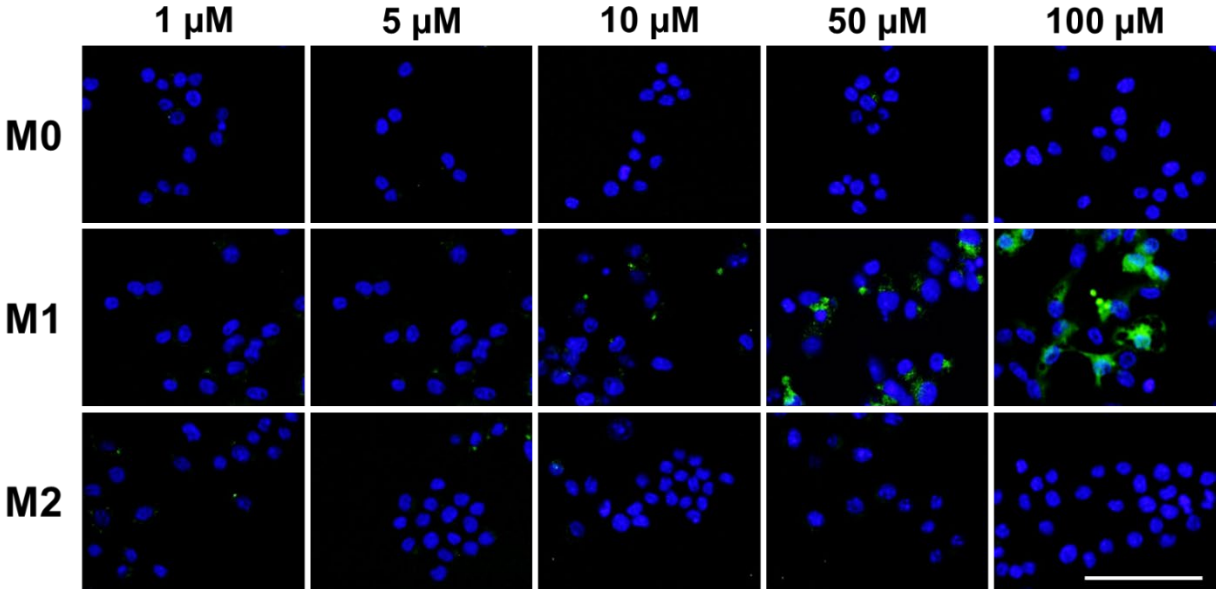

Supplementary Fig. 6. Concentration-dependent selectivity of 2-NBDG in RAW264.7, All the images were acquired at 40x magnification. Each three experiment was repeated independently with similar results. Scale bar, 50 μm.

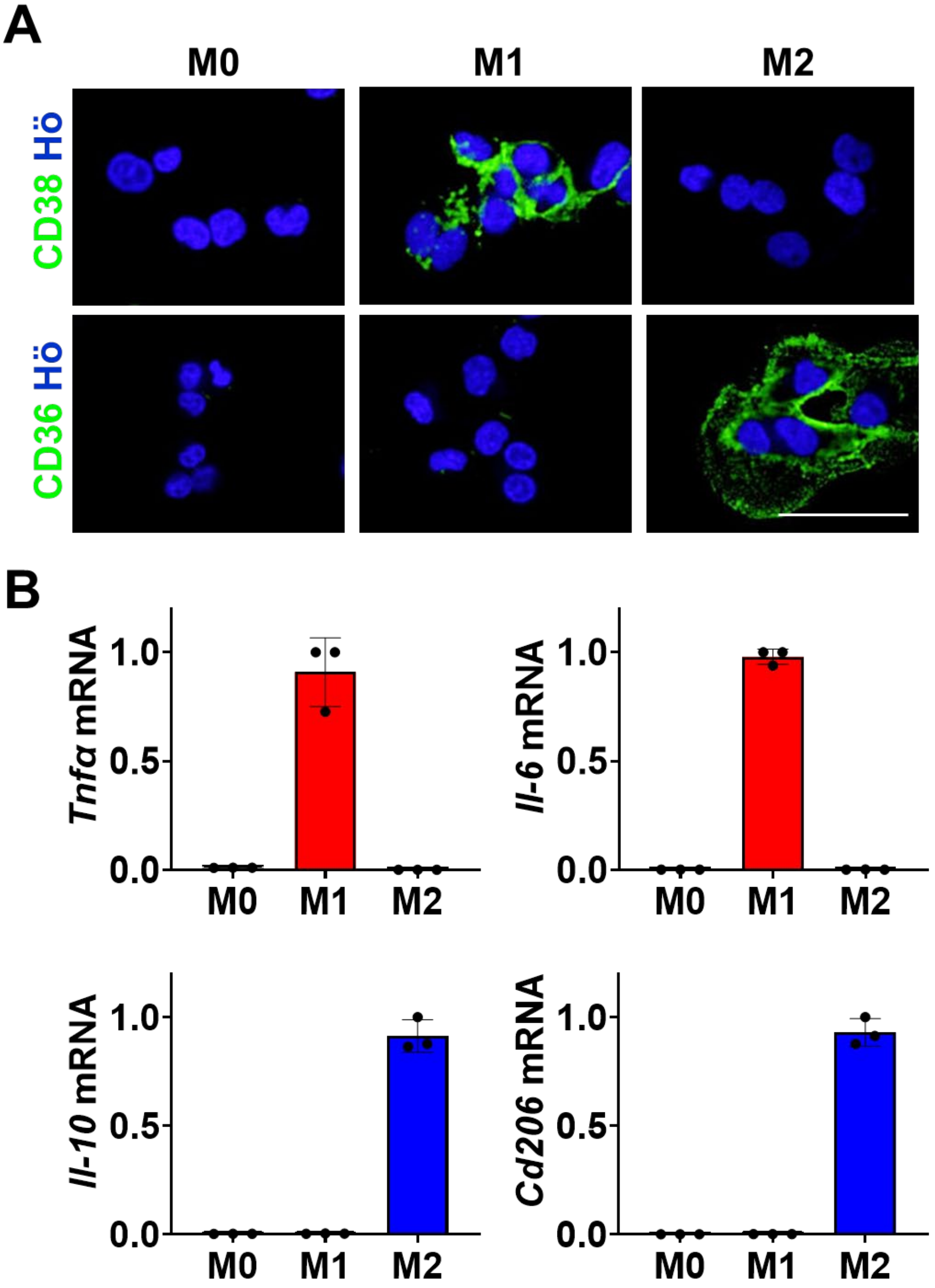

Supplementary Fig. 7. Characterization of polarized macrophages from THP-1 cell by A) images and B) gene expression of specific biomarkers. All the images were acquired at 40x magnification, and the scale par represented 50 μm. Data pooled from three individual experiments. Data are analyzed with 3 samples over 3 independent experiments. All bar graphs show the mean ± SD (n=3). Source data are provided as a Source data file.

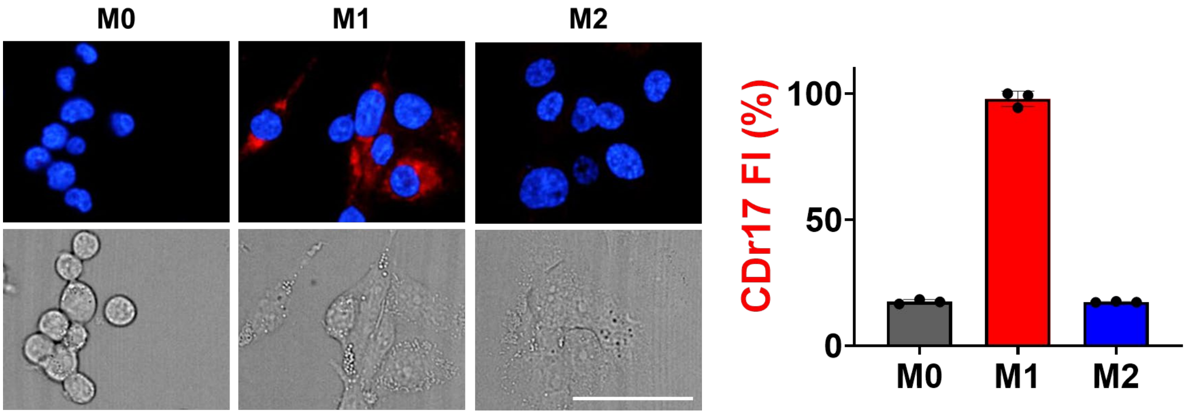

Supplementary Fig. 8. Selectivity of CDr17 in THP-1, and the numeric value of fluorescent intensity (FI) was presented, All the images were acquired at 40x magnification. Data are presented as mean values ± SD (n=3). All error bars represented standard deviation from three independent measurements. Source data are provided as a Source data file. Scale bar, 50 μm.

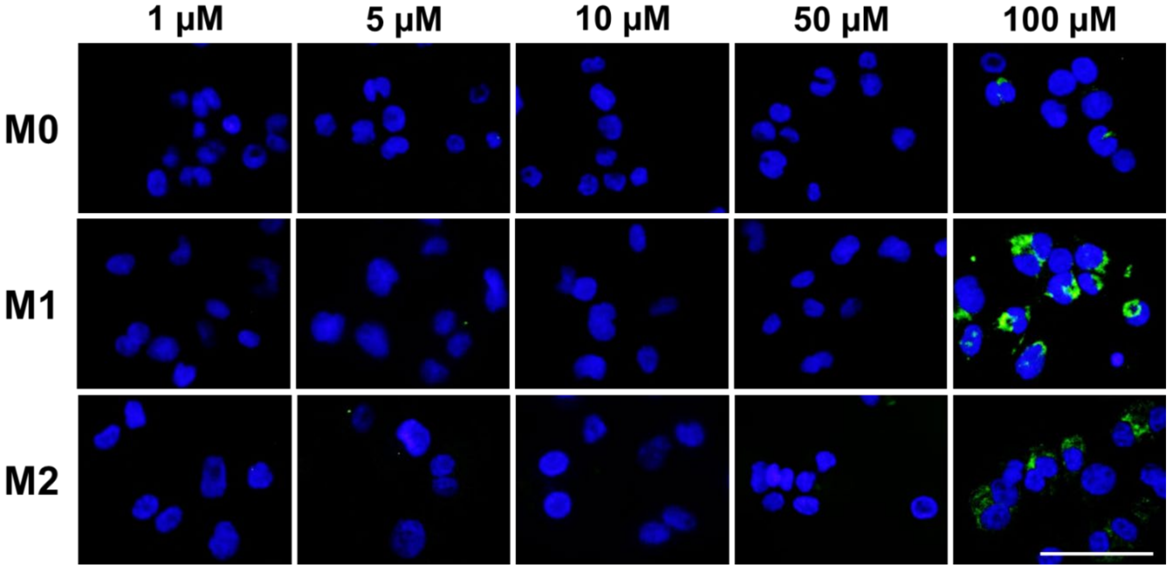

Supplementary Fig. 9. Concentration-dependent selectivity of 2-NBDG in THP-1, All the images were acquired at 40x magnification. Each three experiment was repeated independently with similar results. Scale bar, 50 μm.

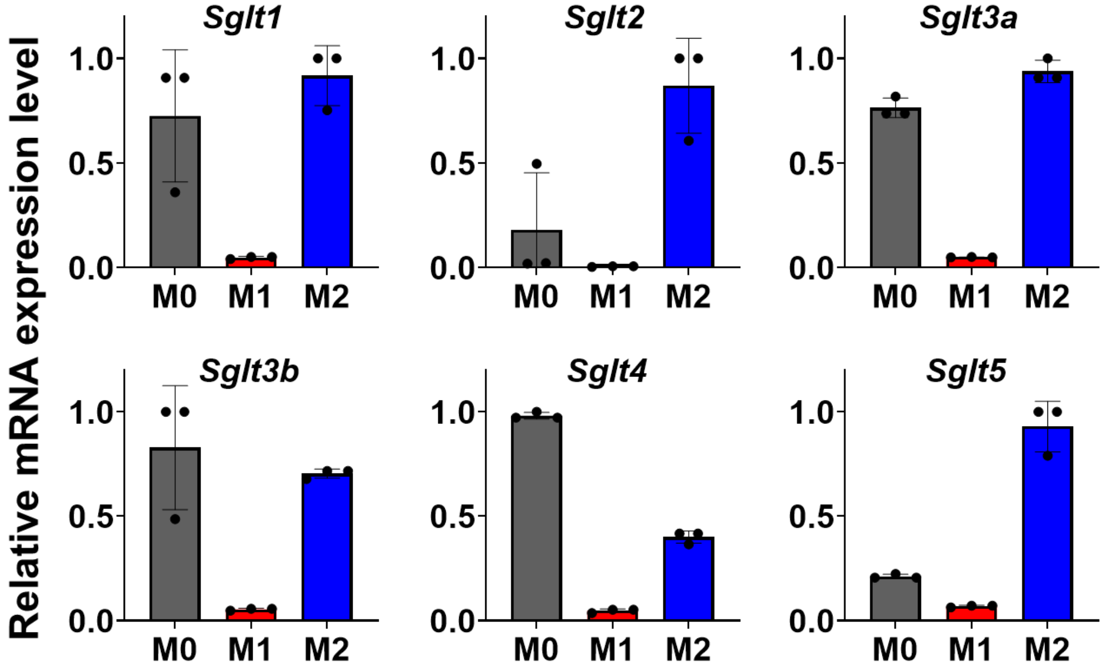

Supplementary Fig. 10. Gene levels of SGLT family in differentiated macrophages from RAW264.7. Data pooled from three individual experiments. Data are analyzed with 3 samples over 3 independent experiments. All bar graphs show the mean ± SD (n=3). Source data are provided as a Source data file.

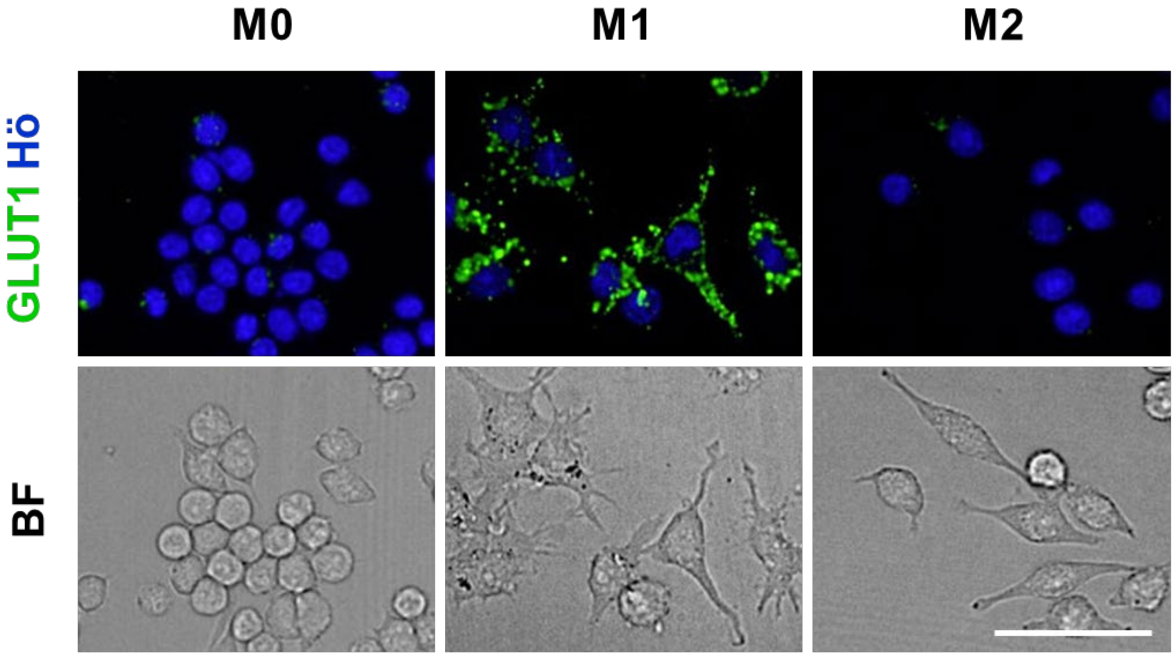

Supplementary Fig. 11. Immunochemistry confirms GLUT1 expression level in differentiated macrophages from RAW264.7. All the images were acquired at 40x magnification. Each three experiment was repeated independently with similar results. Scale bar, 50 μm.

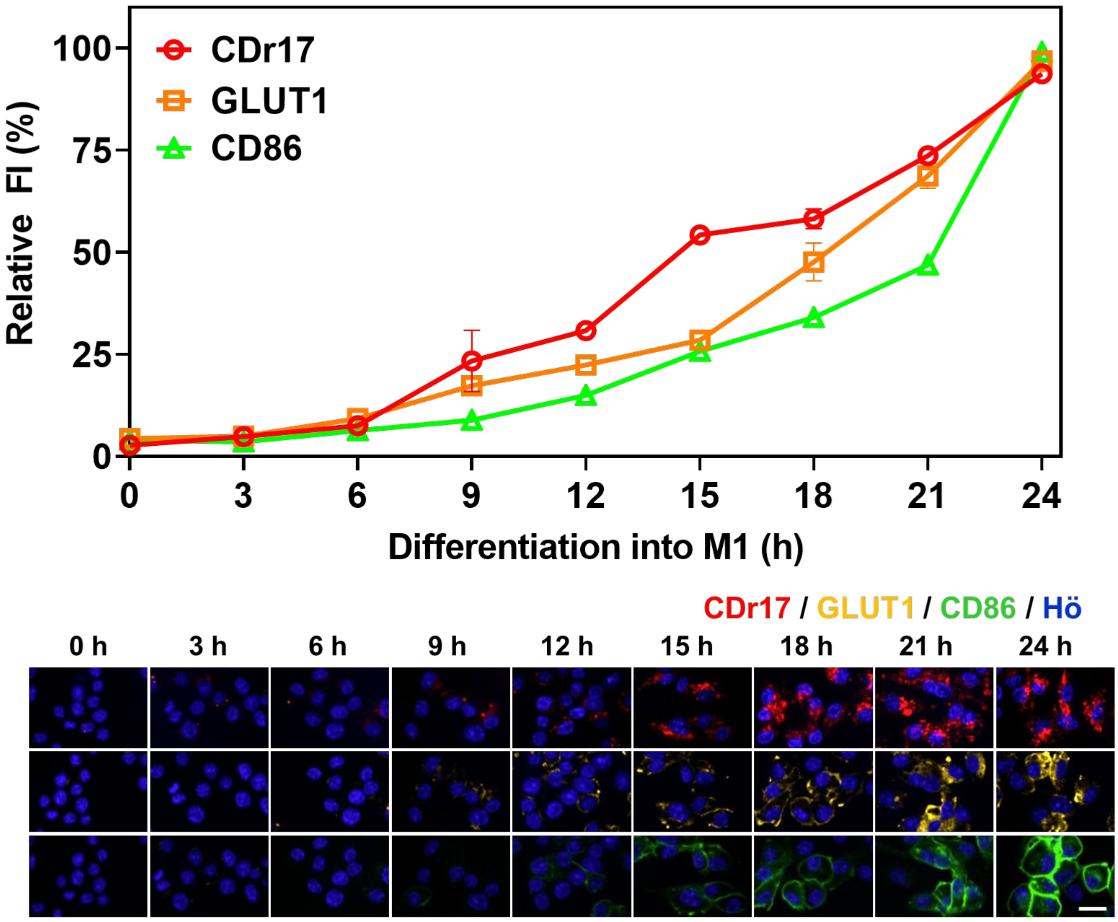

Supplementary Fig. 12. Correlation between M1 polarization, CDr17, and GLUT1. RAW264.7 cells were polarized into M1 macrophages with the treatment of LPS (100 ng/mL) and IFN-γ (20 ng/mL). During polarization, the intensity of CDr17, GLUT1, and CD86 was checked at 3 h intervals until 24 h. The cells were firstly stained with CDr17 (1 μM, 30 min), and were fixed and permeabilized. The fixed cells were incubated with antibodies. All the images were acquired at 40x magnification. The fluorescent intensity (FI) of each factor was obtained and plotted into the graph. Data are presented as mean values ± SD (n=3). Source data are provided as a Source data file. Scale bar, 20 μm.

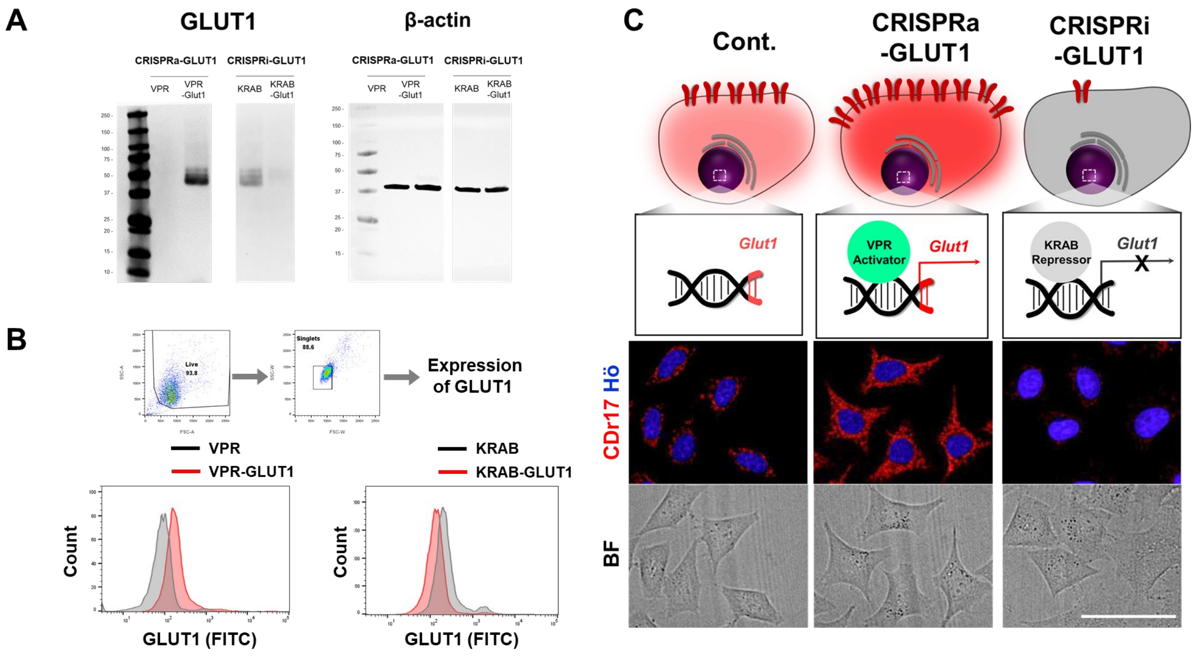

Supplementary Fig. 13. Target validation of CDr17 in CRISPRa-, and CRISPRi-GLUT1 model system. The system was established via CRISPR/dCas9 to activate (CRISPRa) and inhibit (CRISPRi) GLUT1 expression in HeLa cells. A) The protein expression level of GLUT1 was confirmed by western blot, and the samples derived from the same experiment and the gels were processed. B) It was checked double by flow cytometry. The gating strategy (FSC-A/SSC-A) was used to exclude cell debris and aggregates, and (FSC-W/SSC-W) was applied to get the singlets. Each two experiments was repeated independently with similar results. Each three experiments was repeated independently with similar results. C) The intensity of CDr17 (1 μM, 30 min) was confirmed. Scale bar, 50 μm. Source data are provided as a Souce data file.

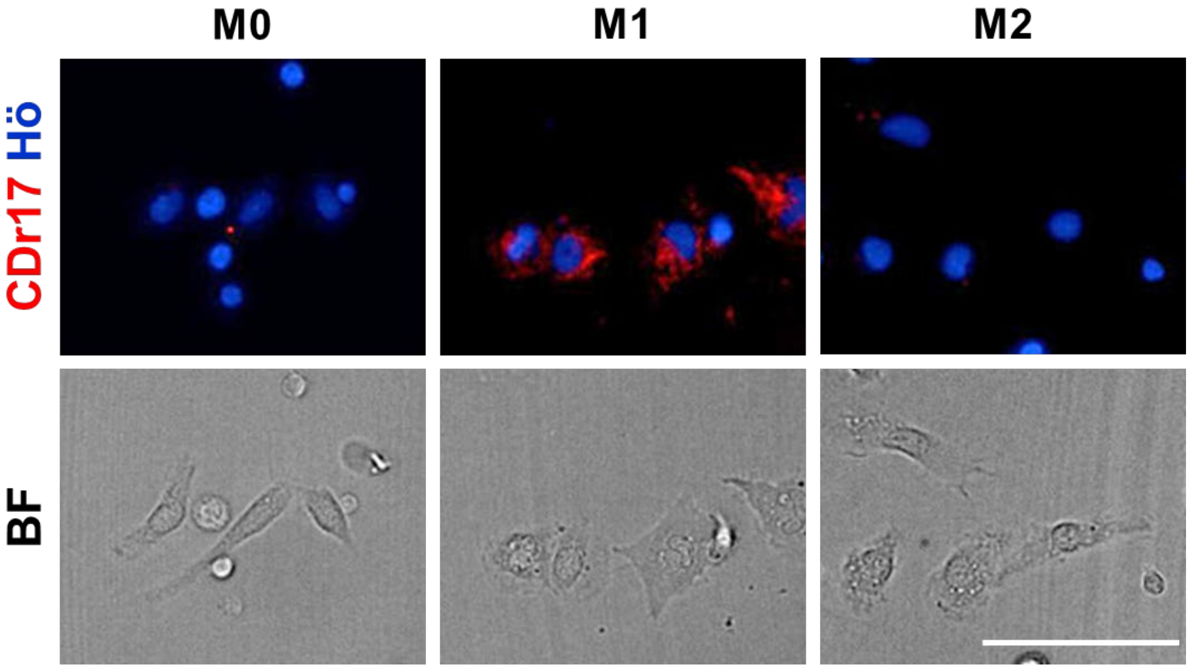

Supplementary Fig. 14. CDr17 selectivity in M0, M1, and M2 macrophages derived from mouse peritoneal macrophages. All the images were acquired at 40x magnification. Each three experiments was repeated independently with similar results. Scale bar, 50 μm.

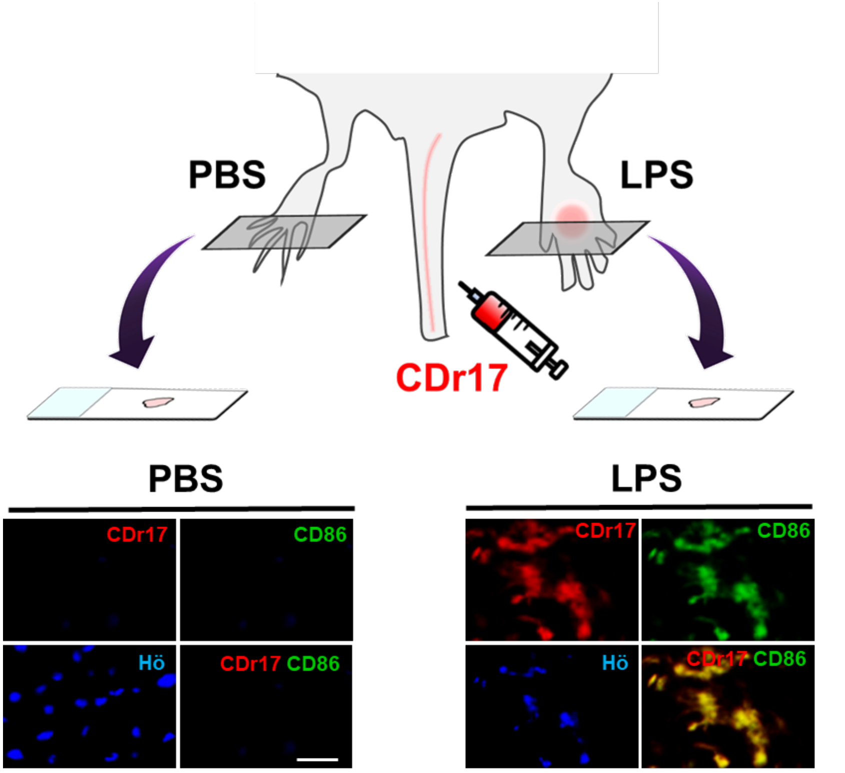

Supplementary Fig. 15. Tissue section images of CDr17 from the acute inflammation-induced animal model after intravenously injection of CDr17. All the images were acquired at 20x magnification. Each three experiments was repeated independently with similar results. Scale bar, 20 μm.

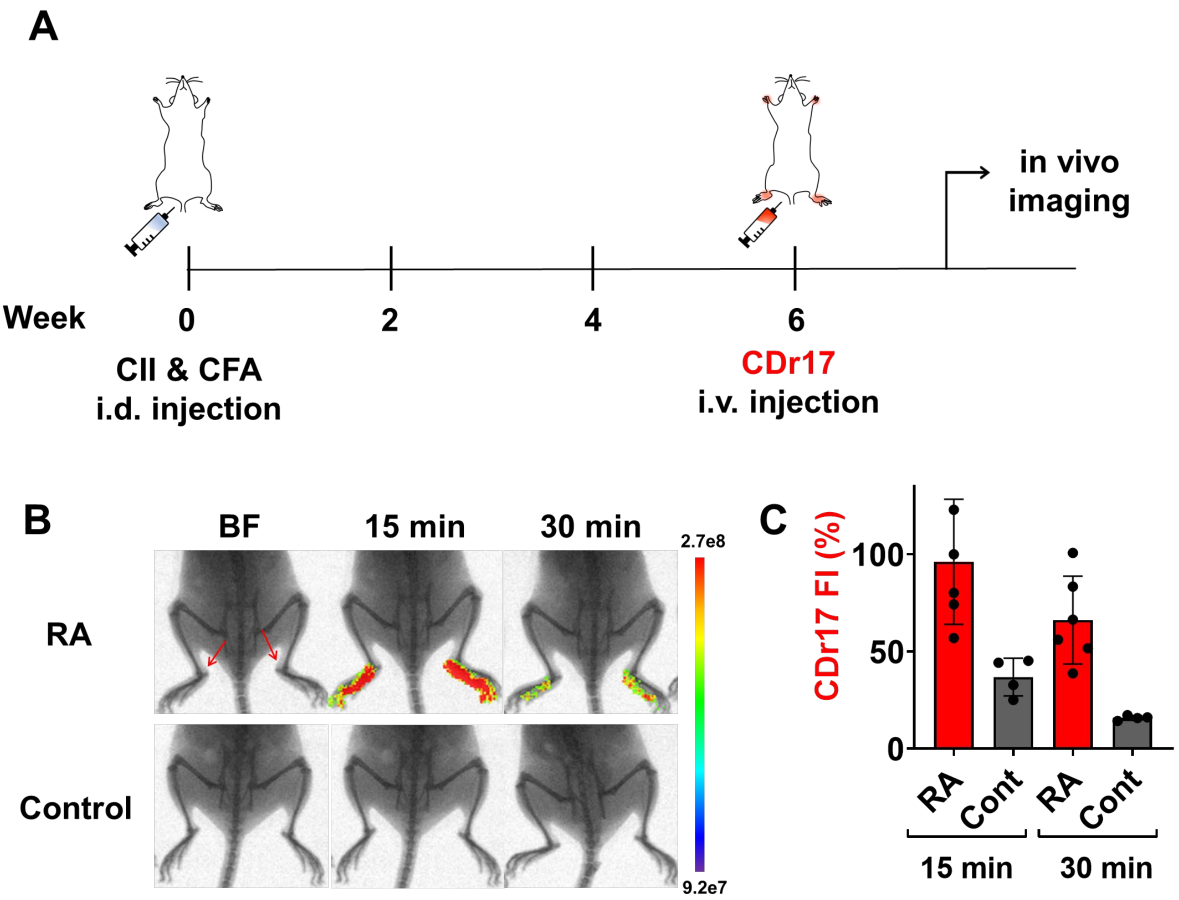

Supplementary Fig. 16. CDr17 selectivity in CIA animals. A) Workflow of collagen-induced arthritis (CIA) model preparation and in vivo imaging. (CII: Collagen Type II; CFA: complete Freund's adjuvant) B, C) The CDr17 fluorescent intensity (FI) from RA and cont animals was displayed. The red arrow represented the swelling joint part. Data are presented as mean ± SD. RA (n = 6), cont (n=4) biologically independent mice per group. Each dot represents an individual mouse. Each three experiment was repeated independently with similar results. Source data are provided as a Source data file.

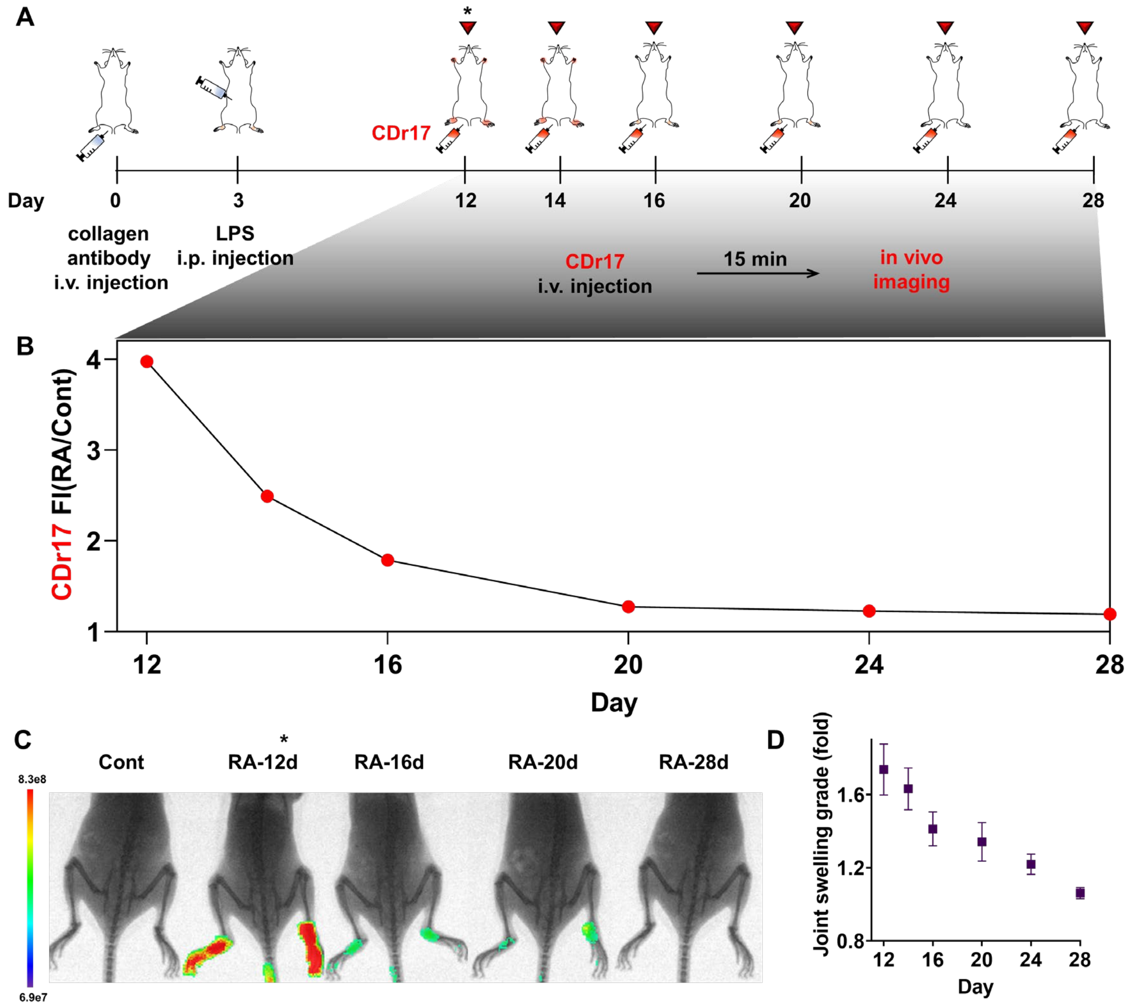

Supplementary Fig. 17. Tracking the CDr17 signal in RA animals over time injection. A) Workflow of collagen antibody-induced arthritis (CAIA) model preparation and in vivo imaging. The star was marked to represent the day for the maximum severity, and the red triangle showed when in vivo images were taken. The CDr17 fluorescent intensity (FI) from RA (n=3) by cont (n=2) animals was displayed in B) the graph, and C) images. D) The joint swelling grade of RA (n=3) was decreased after reaching the maximum score following the days. Data are presented as mean values ± SD. Each two experiment was repeated independently with similar results. Source data are provided as a Source data file.

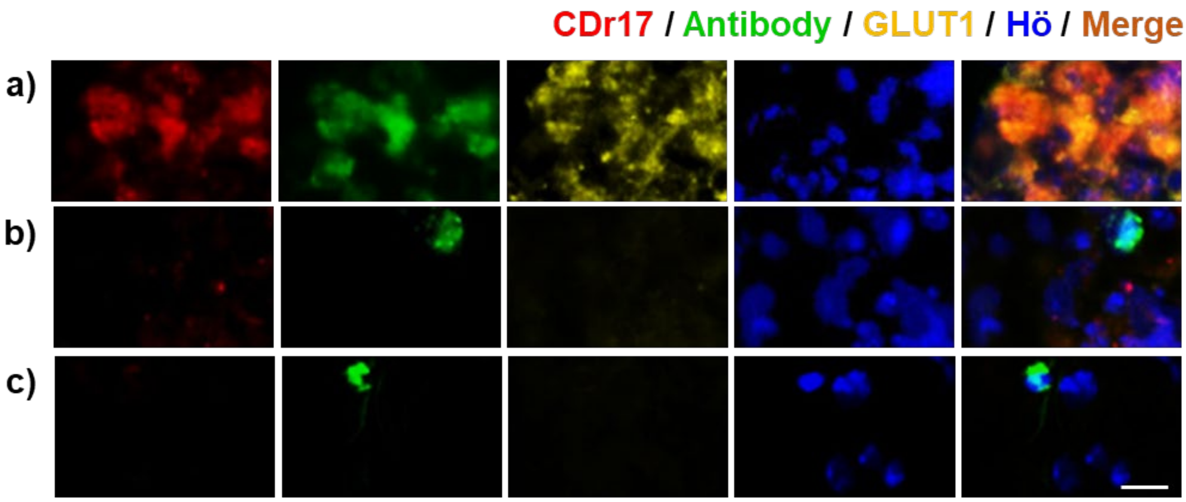

Supplementary Fig. 18. CDr17 specificity to M1 macrophages compared to other cell types in synovium tissues from RA animals. Antibody: a) CD68, b) CD11c, C) CD19. **CDr17** strongly stained CD68-positive cells than CD11c and CD19-positive cells, and they have higher expression levels of GLUT1. Scale bar: 20 μm.

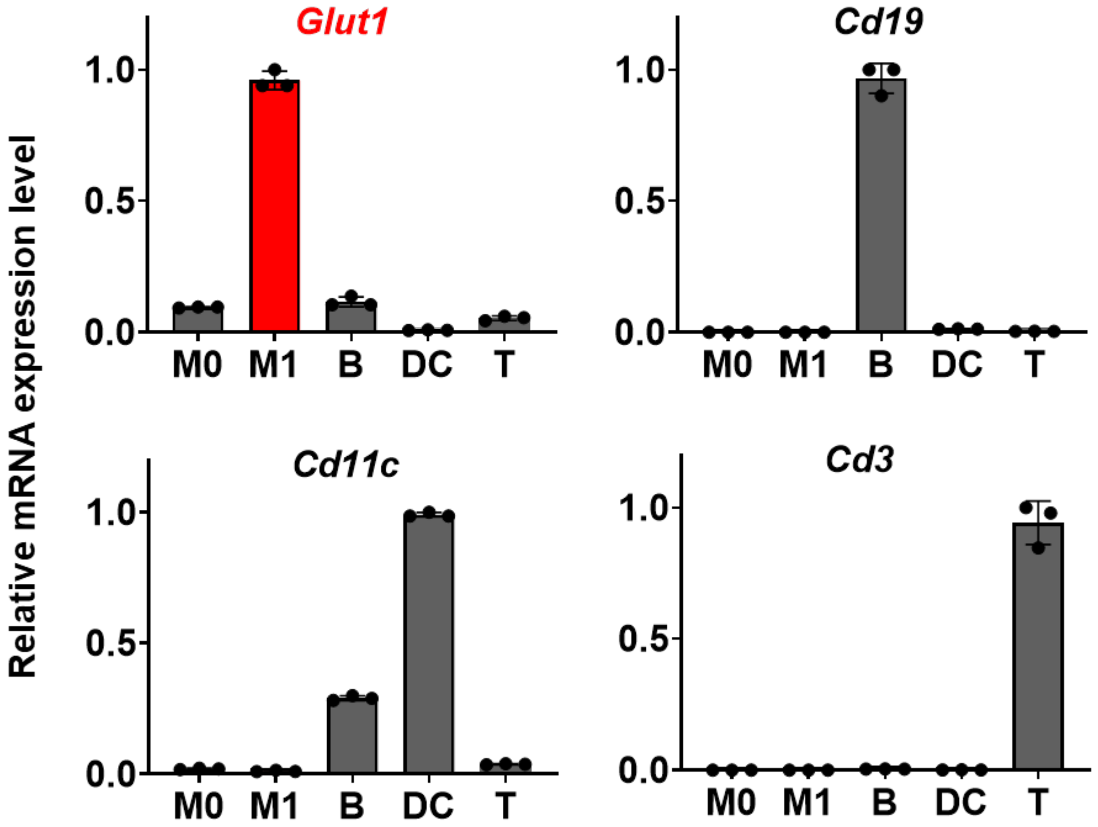

Supplementary Fig. 19. M1 macrophages have higher GLUT1 expression. Each cell type was confirmed with their specific biomarkers (CD19, CD11c, CD3) respectively, and compared GLUT1 level among them. Data pooled from three individual experiments. Data are analyzed with 3 samples over 3 independent experiments. All the bar graphs show the mean ± SD (n=3). Source data are provided as a Source data file.

Supplementary Table 2. Sequences of primers

Species	Gene	Fwd (5' -> 3')	Rev (5' -> 3')
Mouse	mGLUT1	TCAACACGGCCTTCACTG	CACGATGCTCAGATAGGACATC
	mGLUT2	TGTGCTGCTGGATAAATTCGCCTG	AACCATGAACCAAGGGATTGGACC
	mGLUT3	TTCTGGTCGGAATGCTCTTC	AATGTCCTCGAAAGTCCTGC
	mGLUT4	GTAACTTCATTGTCGGCATGG	AGCTGAGATCTGGTCAAACG
	mGLUT5	GGCTCATCTTCCCCTTCATTC	ATGAATGTCCTGCCCTTGG
	mGLUT6	TTGGTGCTGTGAGGCT	TGGCACAAACTGGACGTA
	mGLUT7	GATTCTCCTGCTGTCTGGCTAT	GATGGATGGAAACGTCAACC
	mGLUT8	TTCATGGCCTTTCTAGTGACC	GAGTCCTGCCTTTAGTCTCAG
	mGLUT9	TGCTTCCTCGTCTTCGCCACAATA	CTCTTGGCAAATGCCTGGCTGATT
	mGLUT10	ACCAAAGGACAGTCTTTAGCTG	ATCTTCCAAGCAGACGGATG
	mGLUT12	GGGTGTCAACCTTCTCATCTC	CCAAAGAGCATCCCTTAGTCTC
	mGLUT13	GGCCTGTAAAGATTGGGCCT	AGCTGGTTGTGGTTTCCCAA
	mSLC5A1	AGAGGGGAACAGACAACACA	ACCAAAACCAGGGCATTCCA
	mSLC5A2	TCAGATTCTTTCCAGCCAGG	GCCTGACTCCTATCACCTGC
	mSLC5A4a	GAGAACGGAGCTGATGATAG	GGCTTCTCTGTTGTGTCTGT
	mSLC5A4b	CAGAACCTCCTGAAG	CACCAGCTTCTTCTTGAGTT
	mSLC5A9	GCTTGTGGCAATGGAACC	AACACCAGGTGGCCAAGA
	mSLC5A10	CGCACGTACTTATCTGTCCT	GCAGGGCATCTGTGTATATC
	mTNFα	AAGCCTGTAGCCCACGTCGTA	GGCACCACTAGTTGGTTGTCTTTG
	mIL-6	ACAACCACGGCCTTCCCTACTT	CACGATTTCCCAGAGAACATGTG
	mCD206	GTTCACCTGGAGTGATGGTTCTC	AGGACATGCCAGGGTCACCTTT
	mIL-10	CCAAGCCTTATCGGAAATGA	TTCACAGGGGAGAAATCG
	mCD19	GCCACAGCTTTAGATGAAGGCAC	CATCCACCAGTTCTCAACAGCC
	mCD11c	TGCCAGGATGACCTTAGTGTCG	CAGAGTGACTGTGGTTCCGTAG
	mCD3	ATGGCTACTGCTGTCAGGTCCA	ATGCGGTGGAACACTTTCTGG
	mGAPDH	TGTCCGTCGTGGATCTGAC	CCTGCTTCACCACCTTCTTG
Human	hTNFα	CCAGCTGGAGAAGGGTGAC	AGGCGTTTGGGAAGGTTG
	hIL-6	GGATTCAATGAGGAGACTTGCC	ACAGCTCTGGCTTGTTCCTCAC
	hCD206	ACCTCACAAGTATCCACACCATC	CTTTCATCACCACACAATCCTC
	hIL-10	GACTTTAAGGGTTACCTGGGTTG	TCACATGCGCCTTGATGTCTG
	β-actin	GGATGCAGAAGGAGATCACTG	CGATCCACACGGAGTACTTG

Supplementary methods

Materials and methods for preparation of LC library

All used compounds and solvents were purchased from Alfa Aesar (Haverhill, MA, USA), Sigma Aldrich (St. Louis, MO, USA), Combi-Blocks (San Diego, USA), TCI (Tokyo, Japan), and Samchun Chemicals (Seoul, Republic of Korea). All the chemicals were directly used without further purification. MERCK silica gel 60 (230-400 mesh, 0.040-0.063 mm) was used for normal-phase column chromatography. The optical properties were performed with SpectraMax M2e spectrophotometer (Molecular Devices) in 96 well plate (clear bottom) and QS high-precision cuvette. The relative fluorescence quantum yield method was selected, and Nile Blue (Φ = 0.27) was utilized as the standard. The quantum yield equation was calculated by equation (1). The LC compounds were characterized by LC-MS (Agilent Technology) using a C18 reversed-phase HPLC column (2 μm, 4.6 mm × 50 mm, 100 Å) or (1.8 μm, 2.1 mm × 50 mm, 80 Å). A gradient elution of 10% B in 2 min followed by 100% B in 13 min was used at flow rate of 0.7 mL/min, or 20% B in 2 min followed by 80% B in 4 min at flow rate of 0.3 mL/min (solvent A: H_2O, 0.1% trifluoroacetic acid (TFA); B: acetonitrile, 0.1%TFA). High-performance liquid chromatography (HPLC) was utilized on Prep. HPLC (Shimadzu) with a PDA detector with a C18(2) Luna column (5 μm, 250 mm × 21.2 mm, 100 Å). A gradient elution of 5% B to 100% B for 55 min was used at flow rate of 6 mL/min (solvent A: H_2O, 0.1% trifluoroacetic acid (TFA); B: acetonitrile, 0.1% TFA). ^{1}H and ^{13}C NMR spectra were obtained from Brucker AVANCE III HD 850. The HR-MS measurement (JEOL JMS700) was entrusted to Daegu center in Korea Basic Science Institute (KBSI).

$$\Phi_{fi} = (F_i/F_s)(f_s/f_i)(n_i/n_s)^2 \cdot \Phi_f^s \qquad (1)$$

Where Φ_{fi} and Φ_{fs} represented the fluorescence quantum yield of sample and standard, respectively. F represented the area under curve of the fluorescence spectrum (from 630 to 800 nm), n represented the refractive index of the solvent, and f represented the absorption factor ($f = 1 - 10^{-A}$, where A represented the absorbance) at the excitation wavelength selected for sample and standard.

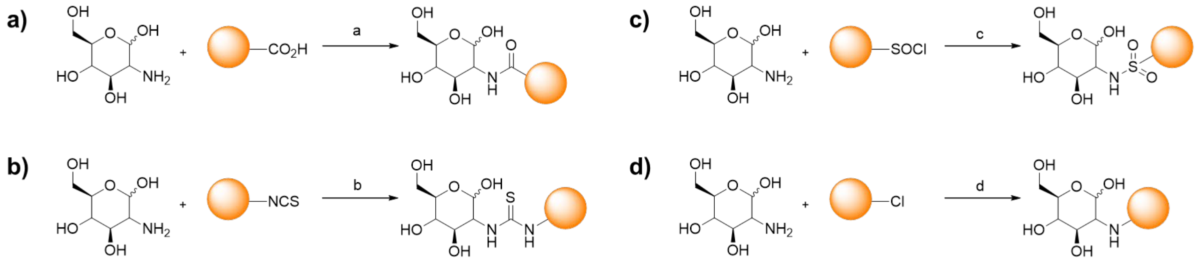

Supplementary Fig. 20. General schematic procedures for LC library. a) HATU, DIEA, DMF, 45℃, 2 h; b) DIEA, MeOH, RT, 2 h; c) DIEA, DMF, 45℃, 2 h; (d) $NaHCO_3$, MeOH, RT, 16 h. The orange circle represents fluorophores.

General procedure of LC library

For LC library construction, supplementary Fig. 20 was used, and it was applied to four carbohydrates backbones. For method a), carbohydrate (3 eq), fluorophore (1 eq), HATU (3 eq), and DIEA (5 eq) were dissolved in DMF. The mixture was stirred at 45℃ for 2 h. For amine and isothiocyanate reaction (method b)), carbohydrate (3 eq), fluorophore (1 eq), and DIEA (10 eq) were dissolved in MeOH. For method c), carbohydrate (3 eq), fluorophore (1 eq), and DIEA (10 eq) were dissolved in DMF.For method d), carbohydrate (3 eq), fluorophore (1 eq), and $NaHCO_3$ (3 eq) were dissolved in MeOH. After the reaction, the volatile was removed under reduced pressure and the residue was purified via silica gel chromatography using a gradient of DCM and MeOH. All the compounds in LC library were confirmed by LC-MS, and it was described in Supplementary Table 2. The hit compound, **CDr17**, was characterized by ^{1}H NMR, ^{13}C NMR, 2D NMR, LC-MS, and HR-MS.

Synthesis of CDr17

The reaction followed General procedure a. Glucosamine (10.7 mg, 0.03 mmole), cy51 (5 mg, 0.01 mmole), HATU (22.8 mg, 0.03 mmole), DIEA (17.4 μL, 0.05 mmole) were dissolved in DMF (5 mL), stirring for 2 h, and the mixture was concentrated in vacuo. The residue was purified via silica gel chromatography using MeOH:DCM=1:15 to MeOH:DCM=1:5, and HPLC was used for the further purification to obtain the blue solid (4.2 mg, 53%). ^{1}H NMR (850 MHz, Methanol-d_4) δ 8.56 (s, 1H), 8.26-8.31 (m, 2H), 7.53 (t, J = 8.3 Hz, 2H), 7.44 (t, J = 7.5 Hz, 2H), 7.34 (d, J = 7.9 Hz, 2H), 7.29 (q, J = 7.4 Hz, 2H), 6.67 (t, J = 12.3 Hz, 1H), 6.33 (dd, J = 6.97, 13.7 Hz, 2H), 5.11 (d, J = 3.4 Hz, $H_{1\alpha}$), 4.58 (d, J = 8.3 Hz, $H_{1\beta}$), 4.14 (t, J = 7.4 Hz, 2H), 4.11 (t, J = 7.5 Hz, 2H), 3.86-3.88 (m, $H_{2\alpha}$), 3.79-3.82 (m, $H_{5\alpha}$), 3.70-3.73 (m, $H_{3\alpha}$, $H_{6\alpha}$), 3.64-3.68 (m, $H_{2\beta}$, $H_{6\beta}$), 3.43 (t, J = 8.7 Hz, $H_{3\beta}$), 3.36-3.38 (m, $H_{4\alpha}$), 3.29-3.31 (m, $H_{4\beta}$, $H_{5\beta}$), 2.34-2.40 (m, 2H), 1.84-1.89 (m, 4H), 1.78-1.82 (m, 2H), 1.75 (s, 6H), 1.74 (s, 6H), 1.08 (t, J = 7.4 Hz,

3H). ^{13}C NMR (214 MHz, Methanol-d_4) δ 174.35, 173.52, 173.23, 154.16, 154.11, 142.26, 142.15, 141.26, 141.22, 128.38, 128.34, 125.25, 124.86, 124.80, 122.00, 110.73, 110.65, 103.03, 102.91, 95.80 ($C_{1\alpha}$), 91.19 ($C_{1\beta}$), 76.69 ($C_{5\beta}$), 74.69 ($C_{3\beta}$), 71.71 ($C_{5\alpha}$), 71.23 ($C_{3\alpha}$), 71.18 ($C_{4\alpha}$), 70.85 ($C_{4\beta}$), 61.43 ($C_{6\beta}$), 61.38 ($C_{6\alpha}$), 57.24 ($C_{2\beta}$), 54.43 ($C_{2\alpha}$), 49.19, 49.14, 47.59, 44.90, 43.32, 34.78, 26.52, 26.39, 22.64, 20.47, 10.11. LC-MS (ESI) $[M]^+$: *m/z* calcd for $C_{39}H_{52}N_3O_6$ 658.4, found: 658.4. The HPLC chromatogram and HR-MS spectra of **CDr17** are in Supplementary Fig. 20. and Supplementary Fig. 21.

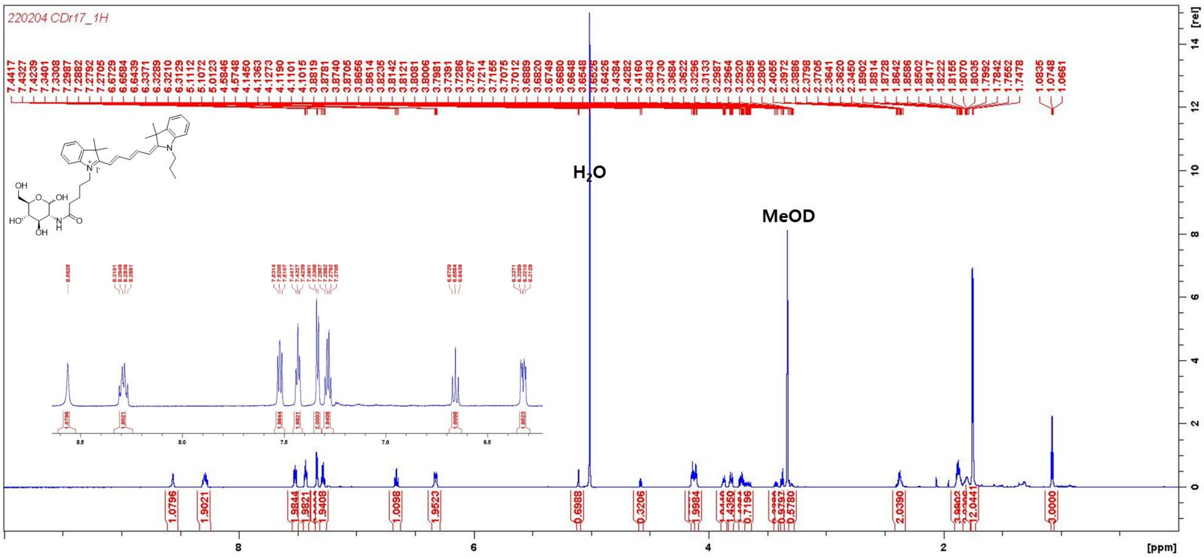

Supplementary Fig. 21. ^{1}H NMR spectrum of **CDr17.**

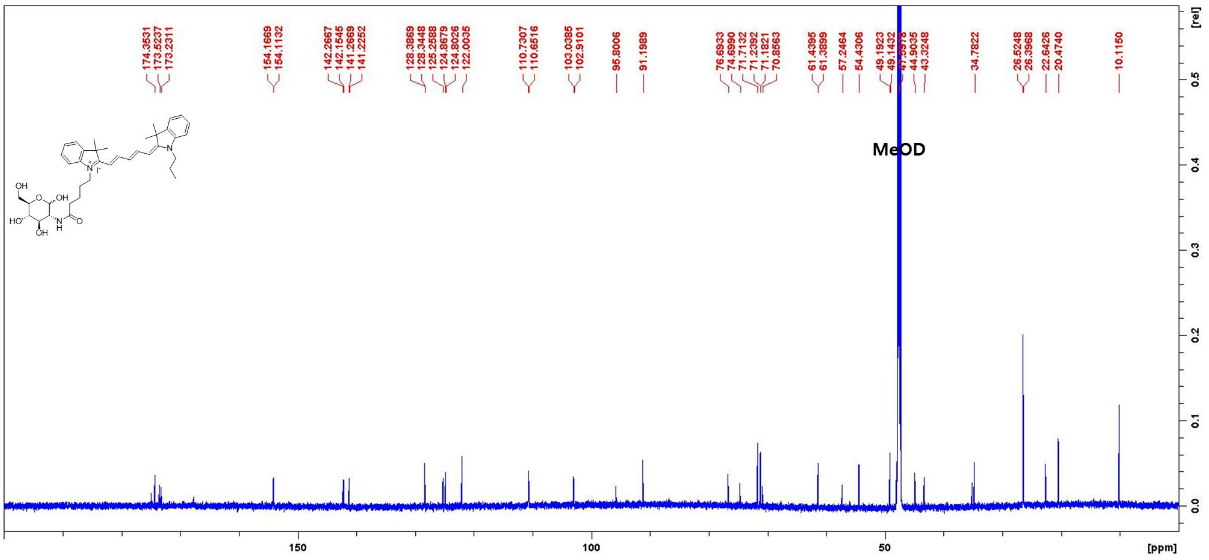

Supplementary Fig. 22. ^{13}C NMR spectrum of **CDr17.**

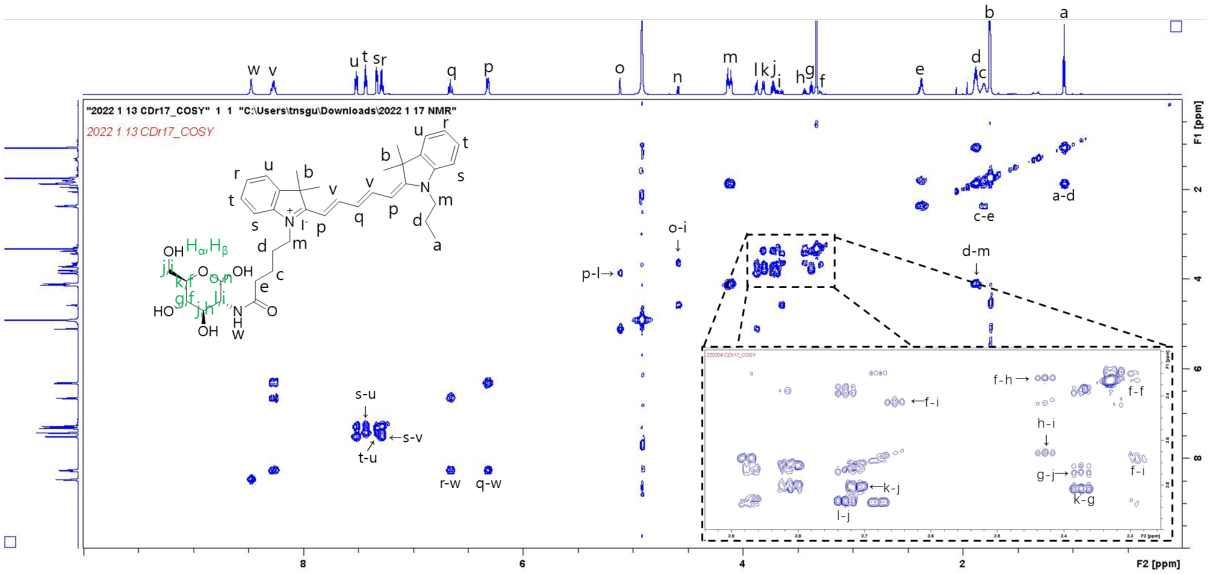

Supplementary Fig. 23. ^{1}H-^{1}H COSY of **CDr17.**

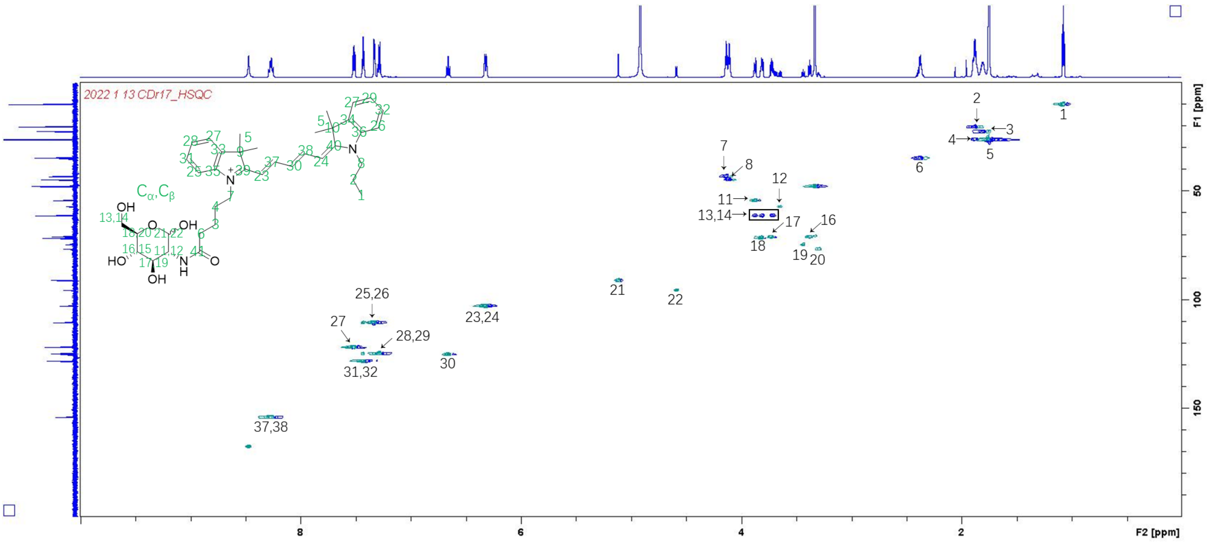

Supplementary Fig. 24. ^{1}H-^{13}C HSQC of **CDr17.**

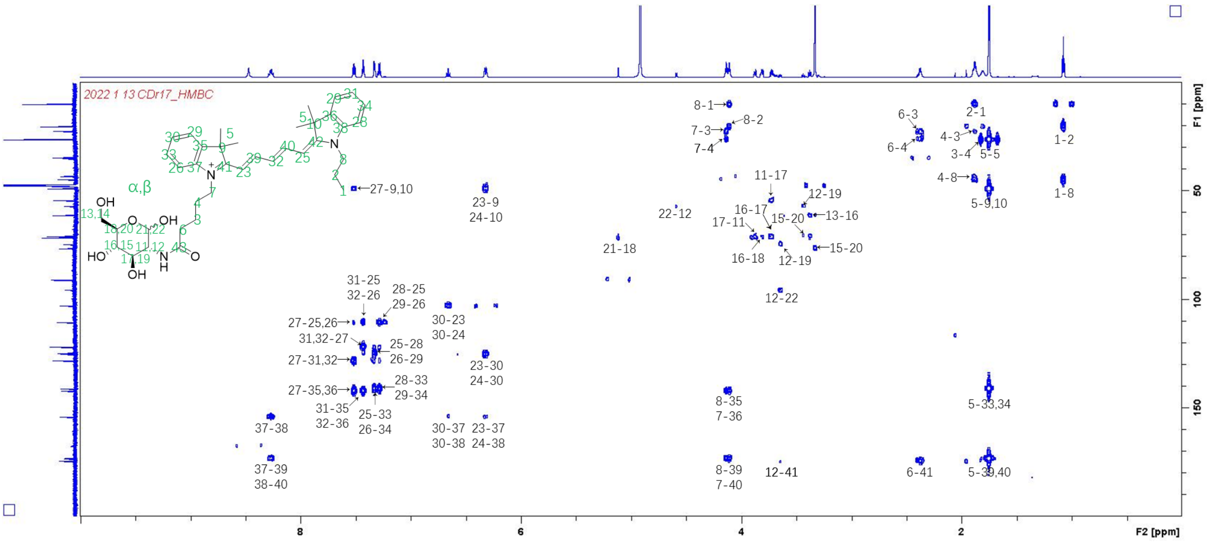

Supplementary Fig. 25. ^{1}H-^{13}C HMBC of **CDr17.**

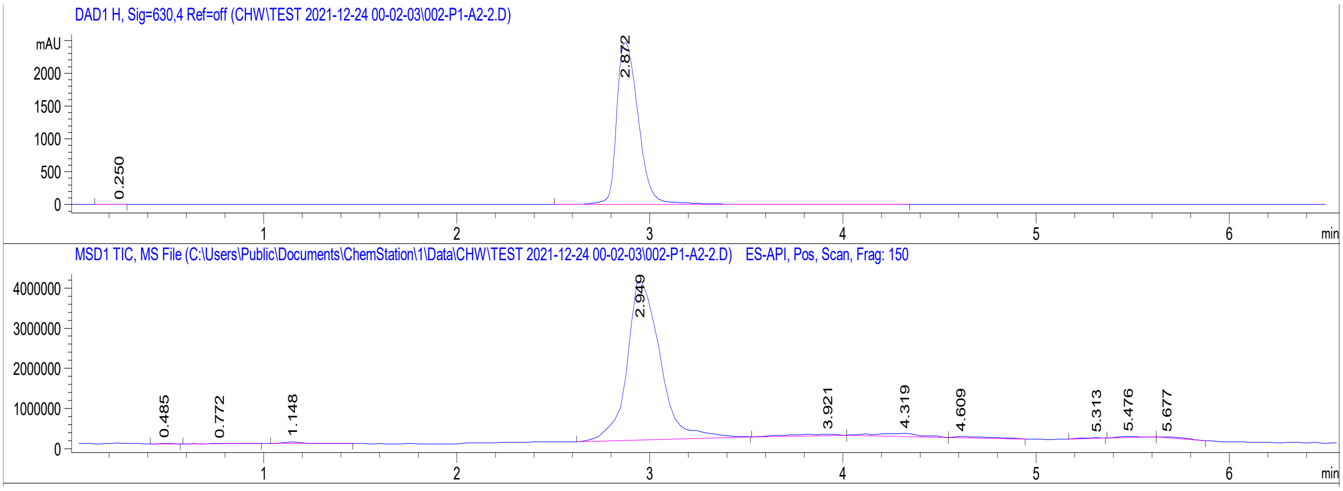

Supplementary Fig. 26. HPLC and mass chromatogram of **CDr17**.

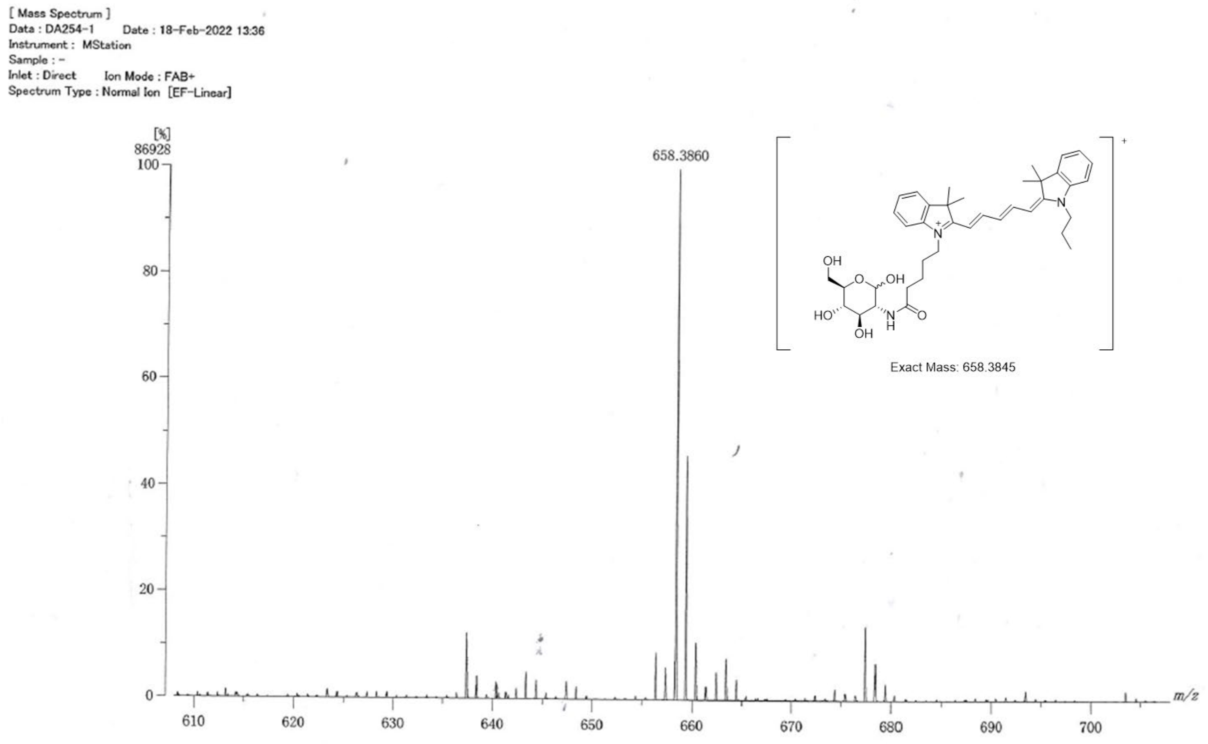

Supplementary Fig. 27. HR-MS spectrum of **CDr17.**

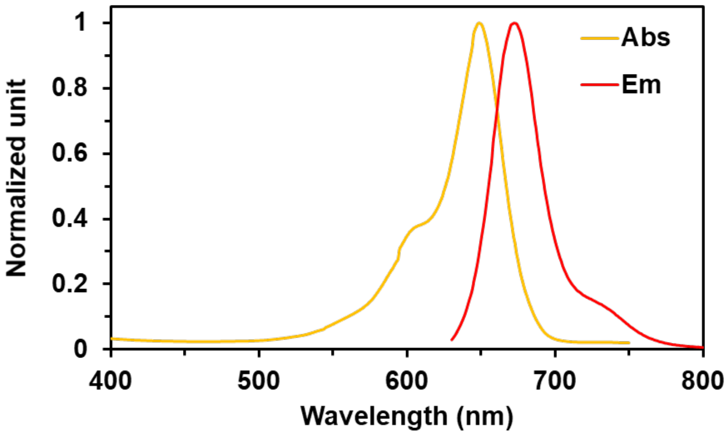

λ_{abs} (nm)	λ_{em} (nm)	ε ($cm^{-1}M^{-1}$)	Φ_{fl}
649	672	190000	0.48

Supplementary Fig. 28. Optical properties of **CDr17** in DMSO. A) Absorbance and emission spectra of **CDr17**. B) Optical properties of **CDr17** in DMSO. The emission spectrum was measured by λ_{ex} = 620 nm.

Supplementary Table 3. Characterization and purity determination of LC compounds by LC-MS

Code	Molecular formula	Exact MS	Observed MS	Purity
A2	$C_{17}H_{20}NO_9$	382.1	382.1	99
A3	$C_{20}H_{27}N_2O_8$	423.2	423.2	99
A4	$C_{16}H_{17}NNaO_8$	352.1	352.1	99
A5	$C_{12}H_{15}N_4O_8$	343.1	343	99
A6	$C_{18}H_{25}N_5NaO_9$	478.1	478.1	99
A7	$C_{18}H_{25}N_2O_7S$	413.1	413.1	95
A8	$C_{24}H_{36}N_3O_8S$	526.2	526.2	73
A9	$C_{27}H_{25}N_2O_{10}S$	569.1	569.2	85
A10	$C_{27}H_{24}NO_{11}$	538.1	538.1	99
A11	$C_{21}H_{28}BFN_3O_6$	434.2	434.2	99
B2	$C_{22}H_{28}BF_2N_3NaO_6$	504.2	504.2	99
B3	$C_{28}H_{32}BFN_3O_7$	552.2	552.3	85
B4	$C_{30}H_{36}BF_2N_3NaO_7$	622.3	622.3	99
B5	$C_{34}H_{38}BF_2N_3O_7$	672.3	672.6	99
B6	$C_{26}H_{31}BF_2N_3O_6$	530.2	530.2	99
B7	$C_{35}H_{46}N_3O_6$	604.3	604.4	99
B8	$C_{36}H_{48}N_3O_9S$	698.3	698.2	99
B9	$C_{39}H_{52}IN_3O_6$	658.4	658.4	99
B10	$C_{38}H_{50}N_3O_9S$	724.3	724.3	99
B11	$C_{40}H_{52}N_3O_9S$	750.3	750.3	95
C2	$C_{17}H_{20}NO_9$	382.1	382.1	99
C3	$C_{20}H_{27}N_2O_8$	423.2	423	90
C4	$C_{15}H_{17}NNaO_9S$	410	410	99
C5	$C_{16}C_{17}NNaO_8$	374.1	374.1	75
C6	$C_{22}H_{25}N_2O_8$	445.2	445.2	99
C7	$C_{12}H_{15}N_4O_8$	343.1	343	99
C8	$C_{18}H_{25}N_5NaO_9$	478.1	478.1	96
C9	$C_{18}H_{25}N_2O_7S$	413.1	413.1	95
C10	$C_{24}H_{36}N_3O_8S$	526.2	526.2	75
C11	$C_{27}H_{25}N_2O_{10}S$	569.1	569	99
D2	$C_{20}H_{26}BF_2N_3NaO_6$	476.2	476.2	99
D3	$C_{22}H_{30}BF_2N_3NaO_6$	504.2	504.2	99
D4	$C_{28}H_{32}BF_2N_3NaO_7$	594.2	594.2	97
D5	$C_{30}H_{36}BF_2N_3NaO_7$	622.3	622.3	99
D6	$C_{34}H_{38}BFN_3O_7$	630.3	630.4	99
D7	$C_{35}H_{46}N_3O_6$	604.3	604.3	99
D8	$C_{36}H_{48}N_3O_9S$	698.3	698.23	99
D9	$C_{39}H_{52}N_3O_6$	658.4	658.4	99
D10	$C_{38}H_{50}N_3O_9S$	724.3	724.3	95

D11	$C_{40}H_{52}N_3O_9S$	750.3	750.3	99
E2	$C_{17}H_{20}NO_9$	382.1	382.1	99
E3	$C_{20}H_{27}N_2O_8$	423.2	423.2	96
E4	$C_{15}H_{17}NNaO_9S$	410	410	99
E5	$C_{16}C_{17}NNaO_8$	374.1	374.1	95
E6	$C_{22}H_{25}N_2O_8$	445.2	445.1	99
E7	$C_{12}H_{15}N_4O_8$	343.1	343	99
E8	$C_{18}H_{25}N_5NaO_9$	478.1	478.1	99
E9	$C_{18}H_{25}N_2O_7S$	413.1	413.1	93
E10	$C_{24}H_{36}N_3O_8S$	526.2	526.2	99
E11	$C_{27}H_{25}N_2O_{10}S$	569.1	569.1	99
F2	$C_{20}H_{26}BF_2N_3NaO_6$	476.2	476.2	99
F3	$C_{22}H_{30}BF_2N_3NaO_6$	504.2	504.2	96
F4	$C_{28}H_{32}BF_2N_3NaO_7$	594.2	594.2	99
F5	$C_{30}H_{36}BF_2N_3NaO_7$	622.3	622.3	99
F6	$C_{34}H_{38}BF_2N_3NaO_7$	672.2	672.2	95
F7	$C_{35}H_{46}N_3O_6$	604.3	604.3	99
F8	$C_{36}H_{48}N_3O_9S$	698.3	698.3	99
F9	$C_{39}H_{52}N_3O_6$	658.4	658.4	86
F10	$C_{38}H_{50}N_3O_9S$	724.3	724.3	99
F11	$C_{40}H_{52}N_3O_9S$	750.3	750.3	95
G2	$C_{17}H_{19}NNaO_9$	404.1	404.1	99
G3	$C_{20}H_{27}N_2O_8$	423.2	423.2	78
G4	$C_{15}H_{17}NNaO_9S$	410	410	95
G5	$C_{16}C_{17}NNaO_8$	374.1	374.1	95
G6	$C_{22}H_{25}N_2O_8$	445.2	445	99
G7	$C_{12}H_{15}N_4O_8$	343.1	343	99
G8	$C_{18}H_{25}N_5NaO_9$	478.1	478.1	78
G9	$C_{18}H_{25}N_2O_7S$	413.1	413.1	95
G10	$C_{24}H_{35}N_3NaO_8S$	548.2	548.2	98
G11	$C_{27}H_{25}N_2O_{10}S$	569.1	569.1	99
H2	$C_{20}H_{26}BF_2N_3NaO_6$	476.2	476.2	99
H3	$C_{22}H_{30}BF_2N_3NaO_6$	504.2	504.1	97
H4	$C_{28}H_{32}BF_2N_3NaO_7$	594.2	594.2	99
H5	$C_{30}H_{36}BF_2N_3NaO_7$	622.3	622.2	99
H6	$C_{34}H_{38}BF_2N_3NaO_7$	672.2	672.2	95
H7	$C_{35}H_{46}N_3O_6$	604.3	604.4	99
H8	$C_{36}H_{48}N_3O_9S$	698.3	698.3	99
H9	$C_{39}H_{52}N_3O_6$	658.4	658.4	99
H10	$C_{38}H_{50}N_3O_9S$	724.3	724.3	99
H11	$C_{40}H_{52}N_3O_9S$	750.3	750.3	95
