# Supplementary material for: Visualizing inflammation with an M1 macrophage selective probe via GLUT1 as the gating target
Source: Nat Commun. 2022 Oct 10;13:5974. doi: 10.1038/s41467-022-33526-z (PMC9550770; doi:10.1038/s41467-022-33526-z)
Supplement: Supplementary file 3 — Reporting Summary [file 41467_2022_33526_MOESM3_ESM.pdf]

nature portfolio

Corresponding author(s): Young-Tae Chang

Last updated by author(s): Aug 30, 2022

Reporting Summary

Nature Portfolio wishes to improve the reproducibility of the work that we publish. This form provides structure for consistency and transparency in reporting. For further information on Nature Portfolio policies, see our Editorial Policies and the Editorial Policy Checklist.

Statistics

For all statistical analyses, confirm that the following items are present in the figure legend, table legend, main text, or Methods section.

n/a	Confirmed	
☐	☒	The exact sample size (*n*) for each experimental group/condition, given as a discrete number and unit of measurement
☐	☒	A statement on whether measurements were taken from distinct samples or whether the same sample was measured repeatedly
☐	☒	The statistical test(s) used AND whether they are one- or two-sided<br>*Only common tests should be described solely by name; describe more complex techniques in the Methods section.*
☒	☐	A description of all covariates tested
☒	☐	A description of any assumptions or corrections, such as tests of normality and adjustment for multiple comparisons
☐	☒	A full description of the statistical parameters including central tendency (e.g. means) or other basic estimates (e.g. regression coefficient) AND variation (e.g. standard deviation) or associated estimates of uncertainty (e.g. confidence intervals)
☒	☐	For null hypothesis testing, the test statistic (e.g. *F*, *t*, *r*) with confidence intervals, effect sizes, degrees of freedom and *P* value noted<br>*Give P values as exact values whenever suitable.*
☒	☐	For Bayesian analysis, information on the choice of priors and Markov chain Monte Carlo settings
☒	☐	For hierarchical and complex designs, identification of the appropriate level for tests and full reporting of outcomes
☒	☐	Estimates of effect sizes (e.g. Cohen's *d*, Pearson's *r*), indicating how they were calculated

Our web collection on statistics for biologists contains articles on many of the points above.

Software and code

Policy information about availability of computer code

Data collection	The cell imaging data were collected from Harmony 4.8 (Perkin Elmer).<br>PCR results were obtained from qPCRsoft 4.0 (analytic jena).<br>Animal imaging was collected from AMI HTX (Spectral Instruments Imaging, Tucson, AZ, USA).
Data analysis	All statiscal analyses were done using Graphpad Prism8.<br>Cell imaging data was analyzed by ImageJ (1.53).<br>The flow cytometry data analysis was done by Flowjo v10.5.0 software.<br>Animal imaging analysis was completed by Aura 4.0 software (Spectral Instruments Imaging).

For manuscripts utilizing custom algorithms or software that are central to the research but not yet described in published literature, software must be made available to editors and reviewers. We strongly encourage code deposition in a community repository (e.g. GitHub). See the Nature Portfolio guidelines for submitting code & software for further information.

Data

Policy information about availability of data

All manuscripts must include a data availability statement. This statement should provide the following information, where applicable:

- Accession codes, unique identifiers, or web links for publicly available datasets
- A description of any restrictions on data availability
- For clinical datasets or third party data, please ensure that the statement adheres to our policy

The authors declare that all data that support our findings in this study are included in the supplemental information and available upon reasonable requests to the corresponding authors. Source Data are provided with this paper.

Human research participants

Policy information about studies involving human research participants and Sex and Gender in Research.

Reporting on sex and gender	N/A
Population characteristics	N/A
Recruitment	N/A
Ethics oversight	N/A

Note that full information on the approval of the study protocol must also be provided in the manuscript.

Field-specific reporting

Please select the one below that is the best fit for your research. If you are not sure, read the appropriate sections before making your selection.

[x] Life sciences [ ] Behavioural & social sciences [ ] Ecological, evolutionary & environmental sciences

For a reference copy of the document with all sections, see nature.com/documents/nr-reporting-summary-flat.pdf

Life sciences study design

All studies must disclose on these points even when the disclosure is negative.

Sample size	1. All the cell experiments repeated three independent measurements to get the statistical analysis.<br>2. All the animal experiments repeated three times with the size of n is over 3 to get the statistical analysis.<br>All sample sizes are listed in the corresponding figure legends.
Data exclusions	No data were excluded from the analyses.
Replication	All experiments were conducted at least three times independently, and similar results were adopted for further analysis to guarantee reproducibility.
Randomization	For in vitro studies, the samples/cells were randomized into different groups prior to treatment. For in vivo experiment, the mice were randomly divided into different groups.
Blinding	Investigators were blinded to group allocation during cell and mice experiments.

Reporting for specific materials, systems and methods

We require information from authors about some types of materials, experimental systems and methods used in many studies. Here, indicate whether each material, system or method listed is relevant to your study. If you are not sure if a list item applies to your research, read the appropriate section before selecting a response.

Materials & experimental systems

n/a	Involved in the study
☐	☒ Antibodies
☐	☒ Eukaryotic cell lines
☒	☐ Palaeontology and archaeology
☐	☒ Animals and other organisms
☒	☐ Clinical data
☒	☐ Dual use research of concern

Methods

n/a	Involved in the study
☒	☐ ChIP-seq
☐	☒ Flow cytometry
☒	☐ MRI-based neuroimaging

Antibodies

Antibodies used

Item / application / company / Cat No / clone / Lot No / Dilution

FITC anti-mouse CD86 Antibody / flow cytometry, immunofluorescence microscopy, immunoprecipitation, immunohistochemical staining / Biolegend / 105006 / GL-1, monoclonal / B301844 / 1:100
APC anti-mouse CD86 Antibody / flow cytometry, immunofluorescence microscopy, immunoprecipitation, immunohistochemical staining / Biolegend / 105012 / GL-1, monoclonal / B268256 / 1:100
anti-mouse CD206 antibody / flow cytometry, immunofluorescence microscopy, immunoprecipitation, immunohistochemical staining / BIO-RAD / MCA2235 / MR5D3, monoclonal / 157637 / 1:100
Human CD38 Alexa Fluor® 488-conjugated Antibody / flow cytometry, immunofluorescence microscopy / R&D Systems / FAB2404G / # 240742, monoclonal / 1563939 / 1:100
CD36 Monoclonal Antibody / flow cytometry, immunofluorescence microscopy, immunoprecipitation, immunohistochemical staining / Invitrogen / MA5-14112 / MA5-14112, monoclonal / VE2997679A / 1:100
GLUT1 Polyclonal Antibody / western blot, immunocytochemistry / Invitrogen / PA1-1063 / polyclonal / UB282436 / 1:1000
Goat anti-Mouse IgG (H+L) Highly Cross-Adsorbed Secondary Antibody, Alexa Fluor Plus 488 / western blot, immunohistochemical staining / Invitrogen / A32723 / polyclonal / VC300588 / 1:500
Goat anti-Rat IgG (H+L) Cross-Adsorbed Secondary Antibody, Alexa Fluor 488 / western blot, immunohistochemical staining / Invitrogen / A-11006 / polyclonal / 2247986 / 1:500
Human Glut1 Antibody / flow cytometry, Cytof, Immunocytochemistry / R&D Systems / MAB1418 / # 202915, monoclonal / IGI0318101 / 1:500
Anti-β-Actin Antibody / western blot / Santa Cruz / sc-47778 / C4, monoclonal / D0618 / 1:1000
Anti-GLUT-1 antibody, Rabbit monoclonal / immunoblotting, immunohistochemistry / Sigma / SAB5500114 / SP168, monoclonal / GR3219906-3
CD19 Monoclonal antibody, Alexa Fluor 488 / immunohistochemistry / eBioscience / 53-0194-82 / 6OMP31, monoclonal / 2106873 / 1:50
CD11c Monoclonal Antibody, Alexa Fluor 488 / immunochistochemistry / eBioscience / 53-0114-82 / N418, monoclonal / 2332618 / 1:50
FITC anti-mouse CD68 Antibody / Intracellular Staining for Flow Cytometry, Flow cytometry / 137006 / FA-11, monoclonal / B308601 / 1:200
GLUT1 Recombinant Rabbit Monoclonal antibody / western blot, immunohistochemistry, immunocytochemistry, flow cytometry / MA5-31960 / SA0377, monoclonal / XD3568681B / 1:200
Goat anti-Rabbit IgG (H+L) Highly Cross-Adsorbed Secondary Antibody, Alexa Fluor Plus 555 / western blot, immunocytochemistry / A32732 / polyclonal / Invitrogen / SE250297 / 1:500

Validation

All antibodies are commercially available and have been routinely validated by manufacturers as described on their websites. All antibodies were tested in the laboratory using postive and negative controls and titrated before all experiments.

Eukaryotic cell lines

Policy information about cell lines and Sex and Gender in Research

Cell line source(s)	RAW 264.7 (KCLB No. 40071), THP-1 (KCLB No. 40202), and HeLa (KCLB No. 10002) were obtained from Korean Cell Line Bank (KCLB).
Authentication	Cell lines were purchased authenticated suppliers via Korean Cell Line Bank. Independent authentication was not done.
Mycoplasma contamination	All cell lines were tested negative for mycoplasma contamination.
Commonly misidentified lines (See ICLAC register)	No commonly misidentified cell lines were used.

Animals and other research organisms

Policy information about studies involving animals; ARRIVE guidelines recommended for reporting animal research, and Sex and Gender in Research

Laboratory animals	C57BL/6J mice; male; 6 to 8 weeks

	BALb/c mice; female; 9 to 10 weeks<br>DBA/1J mice; female; 9 weeks
Wild animals	No wild animals were used in this study.
Reporting on sex	It has been studied that female is preferable to make rheumatoid arthritis models, therefore, we selected female sex for our studies (BALb/c, DBA/1J).<br>For the acute inflammation, we did not consider the sex (C57BL/6J)
Field-collected samples	No field collected samples were used in this study.
Ethics oversight	Animals were provided by the Pohang University of Science and Technology Institutional Care and Use Committe (POSTECH IACUC) (Approval no. 2019-0088). All animal experiments were performed by recommended guidelines.

Note that full information on the approval of the study protocol must also be provided in the manuscript.

Flow Cytometry

Plots

Confirm that:

- [x] The axis labels state the marker and fluorochrome used (e.g. CD4-FITC).
- [x] The axis scales are clearly visible. Include numbers along axes only for bottom left plot of group (a 'group' is an analysis of identical markers).
- [ ] All plots are contour plots with outliers or pseudocolor plots.
- [ ] A numerical value for number of cells or percentage (with statistics) is provided.

Methodology

Sample preparation	For analysis of GLUT1 expression level, each samples were stained with anti-GLUT1 (R&D Systems, MAB1418, 1:500) conjugated with Goat anti-mouse IgG, Alexa Fluor Plus 488 (1:500, Invitrogen, A32723) for 30 min at 37°C . After incubation, cells were washing with PBS twice and harvested.
Instrument	LSR Foretessa 5 laser Flow Cytometer, BD, NJ, USA
Software	Flowjo software was used to collect and anlyze the data.
Cell population abundance	Around 10^5 cells were prepared for each sample, and 5x10^3 cells were analyzed by Flow cytometer.
Gating strategy	The gating strategy (FSC-A/SSC-A) was used to exclude cell debris and aggregates, and (FSC-W/SSC-W) was applied to get the singlets.

- [x] Tick this box to confirm that a figure exemplifying the gating strategy is provided in the Supplementary Information.
